# Isolation and Quantification of Ginsenoside Rh23, a New Anti-Melanogenic Compound from the Leaves of *Panax ginseng*

**DOI:** 10.3390/molecules23020267

**Published:** 2018-01-29

**Authors:** Dae Young Lee, Hyoung-Geun Kim, Yeong-Geun Lee, Jin Hee Kim, Jae Won Lee, Bo-Ram Choi, In-Bae Jang, Geum-Soog Kim, Nam-In Baek

**Affiliations:** 1 Department of Herbal Crop Research, National Institute of Horticultural and Herbal Science, RDA, Eumseong 27709, Korea; dylee0809@gmail.com (D.Y.L.); jaewon3@gmail.com (J.W.L.); bmcbr@korea.kr (B.-R.C.); ikanet@korea.kr (I.-B.J.); kimgs0725@korea.kr (G.-S.K.); 2 Department of Oriental Medicine Biotechnology, Kyung Hee University, Yongin 17104, Korea; zwang05@naver.com (H.-G.K.); lyg629@nate.com (Y.-G.L.); 3 College of Herbal Bio-industry, Daegu Haany University, Gyeongsan 38610, Korea; gonogo1@nate.com

**Keywords:** ginsenoside Rh23, *Panax ginseng*, NMR, UPLC-QTOF/MS, zebrafish, quantitative analysis

## Abstract

A new ginsenoside, named ginsenoside Rh23 (**1**), and 20-*O*-β-d-glucopyranosyl-3β,6α,12β,20β,25-pentahydroxydammar-23-ene (**2**) were isolated from the leaves of hydroponic *Panax ginseng*. Compounds were isolated by various column chromatography and their structures were determined based on spectroscopic methods, including high resolution quadrupole/time of flight mass spectrometry (HR-QTOF/MS), nuclear magnetic resonance (NMR) spectroscopy, and infrared (IR) spectroscopy. To determine anti-melanogenic activity, the change in the melanin content in melan-a cells treated with identified compounds was tested. Additionally, we investigated the melanin inhibitory effects of ginsenoside Rh23 on pigmentation in a zebrafish in vivo model. Compound **1** inhibited potent melanogenesis in melan-a cells with 37.0% melanogenesis inhibition at 80 µM and also presented inhibition on the body pigmentation in zebrafish model. Although compound **2** showed slightly lower inhibitory activity than compound **1**, it also showed significantly decreased melanogenesis in melan-a cell and in zebrafish model. These results indicated that compounds isolated from hydroponic *P. ginseng* may be used as new skin whitening compound through the in vitro and in vivo systems. Furthermore, this study demonstrated the utility of MS-based compound **1** for the quantitative analysis. Ginsenoside Rh23 (**1**) was found at a level of 0.31 mg/g in leaves of hydroponic *P. ginseng*.

## 1. Introduction

*Panax ginseng* C.A. Meyer is a very famous traditional medicinal herb in Asian countries. *Panax* originates from a “panacea”, which means cure-all for diseases. *P. ginseng* is a perennial herbaceous plant belonging to the Araliaceae family [1]. Four- to six-year-old roots of *P. ginseng* are mainly used for therapeutic purposes. Flowers bloom in June, and the leaves, having a palmate shape, of. *P. ginseng* have been mainly cultivated in East Asia, including Korea, China, and Japan [2]. To date, many studies have been reported about the chemical constituents of ginseng roots, leaves, and berries; more than 100 kinds of ginsenosides have been isolated. Various bioactivities of *P. ginseng* were reported, such as the enhancement of immunomodulatory activity, nutritional fortification, improvements of liver function, anti-diabetes, anti-cancer, anti-apoptotic, and anti-oxidant activities [3,4,5,6,7,8,9,10].

Currently, the interest in well-being-related agricultural products of high quality is gradually increasing, leading to hydroponic cultivation of ginseng. Hydroponic cultivation has the advantages of simple cultivation process and shorter growth period than soil cultivation. Hydroponic ginseng needs only 2–4 months in a system that controls temperature, pesticide-free, moisture, light, organic ingredients, etc. [11]. The leaves of soil cultivation ginseng are not used for medicinal purpose and functional vegetables, while the leaves of hydroponic ginseng can be used. In previous study, the contents and composition of ginsenosides in different parts, such as leaves, roots, and fruits of ginseng, were investigated after short-term hydroponic system [12]. The total ginsenoside content of the ginseng leaves was found to be significantly higher at 15.30%, while the content of the ginseng roots at 1.27%. Additionally, the contents of the major ginsenosides components produced in the ginseng leaves cultured in the hydroponic system were observed in the order of: Rg1 > Rd > Re > Rc > Rb2 > Rg2 > Rb1 > Rh1 > Rf [11]. Conclusively, hydroponic conditions led to high ginsenoside content, and the total ginsenoside content in leaves was significantly higher than in roots, suggesting ginseng leaves might be a good source as functional vegetables and medicinal herbs.

Several the known whitening compounds, such as arbutin and kojic acid, have been investigated for their effectiveness in reducing melanogenesis [13]. Unfortunately, it is clearly necessary to find safer and more effective skin-whitening agents, due to the carcinogenic potential of kojic acid and both the safety and side-effects of arbutin [14]. Thereby, a great deal of attention has continuously investigated on the development of new natural products in the cosmetics industry [15,16]. Several studies have reported the inhibition of melanin synthesis from *P. ginseng* grown in soil [17,18,19,20]. However, these studies were reported with well-known compounds, such as cinnamic acid and phenolic compounds, and whitening activity has not been reported from the leaves of hydroponic *P. ginseng* (HPGL). Our ongoing work led to the isolation of minor ginsenosides from HPGL. Usually, ginsenosides in minor or trace amounts cannot be detected with HPLC. Otherwise, the analytical time is very long, which is not convenient for qualifying the ginsenosides in ginseng spices [21,22]. To rapidly quantify novel compounds, a rapid and sensitive method, which can thoroughly detect the trace amounts of novel compounds, should be established. In the present study, a sensitive ultrahigh performance liquid chromatography coupled with quadrupole/time of flight mass spectrometry (UPLC-QTOF-MS) method was established to quantify of new compound.

Based on the above description, in this work, isolation and identification of a new compound, including physical properties and quantification, were revealed by spectroscopic methods, and their anti-melanogenic activities were investigated through in vitro and in vivo systems.

## 2. Results and Discussion

Leaves of hydroponic *Panax ginseng* (HPGL) were extracted with aqueous MeOH and partitioned into ethyl acetate (EtOAc), *n*-butanol (*n*-BuOH), and H_2_O fractions, respectively. Repeated SiO_2_ and ODS column chromatographies of the *n*-BuOH fraction afforded one new ginsenoside (**1**), and one rare ginsenoside (**2**) was isolated from the EtOAc fraction of HPGL.

**Figure 1:**
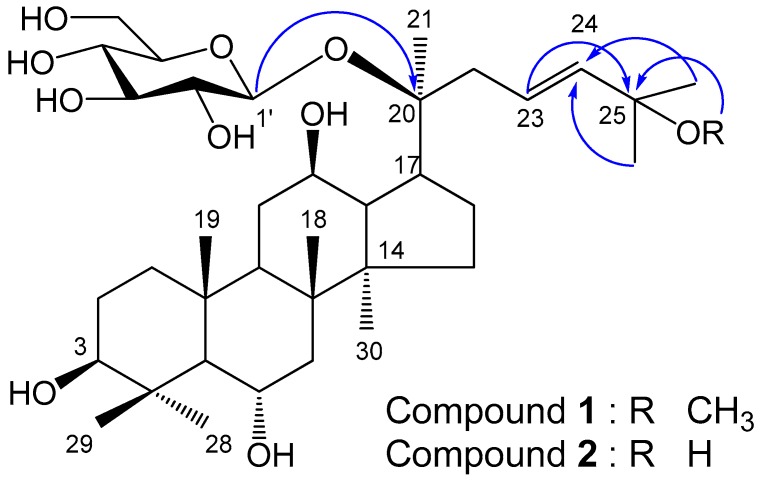
Chemical structures of compounds **1** and **2**, and key gHMBC (arrow) correlations of **1**.

Compound **1**, white powder (methanol), showed a purple color on the TLC, by spraying 10% H_2_SO_4_ and heating. The molecular formula was determined to be C_37_H_64_O_10_ from the quasi-molecular ion peak *m*/*z* 713.44723 [M + COOH]^−^ in the negative QTOF/MS. IR spectrum suggested the presence of hydroxyl group (3377 cm^−1^) and the double bond (1647 cm^−1^). The ^1^H-NMR spectrum (Table 1 and Supplementary Materials) showed two olefin methine proton signals (δ_H_ 6.02, 5.64), three oxygenated methine proton signals (δ_H_ 4.38, 4.03, 3.49), one methoxy proton signal (δ_H_ 3.18), and eight singlet methyl proton signals (δ_H_ 1.94, 1.55, 1.43, 1.33, 1.31, 1.14, 1.04, 0.92), indicating compound **1** has a tetracyclic triterpene moiety including one double bond with trans conformation, and three hydroxyl groups. Additionally, compound **1** is confirmed to be a protopanaxatriol (PPT)-type from the chemical shift of a methyl proton signal at δ_H_ 1.94 (H-28). The chemical shift of H-28 in protopanaxadiol (PPD)-type is usually observed at ca δ_H_ 1.30 [23]. Furthermore, a hemiacetal proton signal (δ_H_ 5.15), and several oxygenated methine and methylene proton signals at δ_H_ 4.45–3.95 were observed as the signals of a sugar moiety. From the coupling constant of the anomer proton signal (*J* = 7.6 Hz), both the hemiacetal proton and H-2 of the sugar moiety were in an axial arrangement. The combination of the above-mentioned data concluded compound **1** to be a protopanaxatriol monoglycoside. The ^13^C-NMR spectrum exhibited 37 carbon signals due to triterpene, methoxy, and hexose moieties. Two olefin methine carbons (δ_C_ 138.5 (C-24), 126.8 (C-23)), one oxygenated quaternary carbon (δ_C_ 74.9 (C-25)), three oxygenated methine carbons (δ_C_ 78.5 (C-3), 70.3 (C-12), 67.7 (C-6)), one methoxy carbon (δ_C_ 50.2 (25-OCH_3_)), and eight methyl carbons (δ_C_ 31.9 (C-28), 26.3 (C-27), 26.1 (C-26), 23.0 (C-21), 17.6 (C-18), 17.4 (C-19), 17.3 (C-30), 16.4 (C-29)) signals were observed for the aglycon moiety. The NMR data of compound **1** were similar to those of compound **2**, with the exception of the chemical shift for an oxygenated quaternary carbon, i.e., C-25. Additionally, the sugar was identified as a β-glucopyranose from the carbon signals hemiacetal (δ_C_ 98.3, (C-1′)), four oxygenated methines (δ_C_ 78.9 (C-3′), 78.2 (C-5′), 75.2 (C-2′), 71.6 (C-4′)), and one oxygenated methylene (δ_C_ 63.0 (C-6′)). In the gradient heteronuclear multiple bond correlation (gHMBC) spectrum, a long-range correlation was observed between the anomeric proton signal (δ_H_ 5.15 (H-1′)) and the oxygenated quaternary carbon signal of the aglycon (δ_C_ 83.0 (C-20)), indicating that the β-glucopyranose were linked to the hydroxyl of C-20 (Figure 1). In addition, the correlation between the methoxy proton signal (δ_H_ 3.18 (25-OCH_3_)) and the oxygenated quaternary carbon signal (δ_c_ 74.9 (C-25)) indicated that the methoxy was linked to C-25 (Figure 1). Based on the above data, the chemical structure of **1** was determined to be 20-*O*-β-d-glucopyranosyl-3β,6α,12β,20β-tetrahydroxy-25-methoxydammar-23-ene, and named as ginsenoside Rh23. Compound **2** was identified to be 20-*O*-β-d-glucopyranosyl-3β,6α,12β,20β,25-pentahydroxydammar-23-ene from the comparisons of NMR and MS data with those reported in the literature [23,24] (Figure 1). Compounds **1** and **2** were, for the first time, isolated from HPGL.

**Figure 2:**
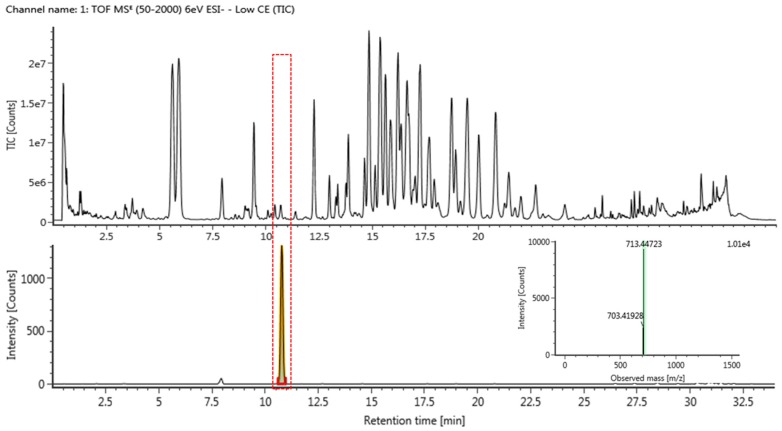
TIC of HPGL extract and ginsenoside Rh23 by UPLC-QTOF/MS in negative-ion mode by selected ion monitoring, and representative QTOF/MS chromatograms

The purity of ginsenoside Rh23 was determined to be more than 99% by the normalization of the peak areas detected by UPLC analysis. Since UPLC-QTOF/MS has been proven to be a suitable tool for the identification of ginsenoside Rh23, the separation of constituents in HPGL extract was performed by UPLC-QTOF/MS in negative-ion mode. Figure 2 shows a typical total ion chromatogram (TIC) of ginsenoside Rh23 and extract with mass detection.

A linear calibration curve was obtained for ginsenoside Rh23 at different concentration levels. The characteristics of the calibration plots are summarized in Table 2. As seen in the table, ginsenoside Rh23 shows excellent correlation coefficients. Detector counts (relative peak area) were linearly dependent on the sample concentration over the range of 0.02–0.8 µg/mL for ginsenoside Rh23. The LODs of ginsenoside Rh23 was 0.002 ppm. The LOQs of ginsenoside Rh23 was determined to be 0.005 ppm by UPLC-QTOF/MS in negative-ion mode. The amount of ginsenoside Rh23 in the HPGL obtained using validation methods (Table 2) was 0.319 mg/g.

**Figure 3:**
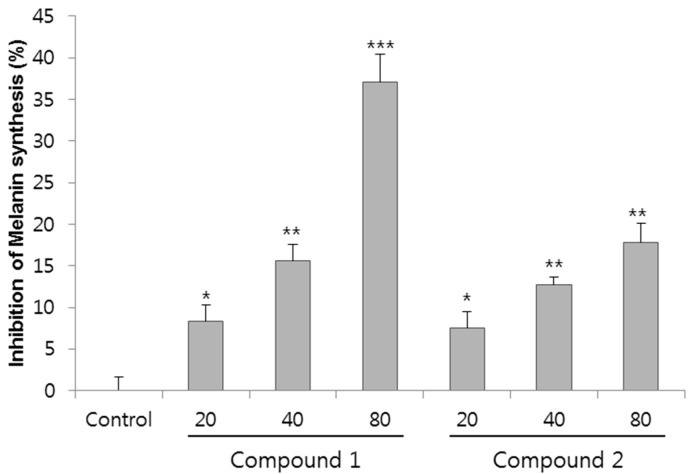
Effects of compounds **1** and **2** on melanogenesis in melan-a cells. The cells were cultured with 0–80 µM of eight compound for four days. Inhibition of melanin synthesis was measured with triplicate experiments. Data are expressed a means ± SD of triplicate determinations. * *p* < 0.05, ** *p* < 0.01, *** *p* < 0.001 versus the control group.

To determine anti-melanogenic activity, the change in the melanin content in melan-a cells treated with purified and identified compounds was studied. Melan-a cells were treated for 72 h with compounds **1** and **2** at concentrations ranging from 0 to 80 µM, and cell viability was assessed via CCK-8 cell viability assay kit. The cell viability of compound **1** and **2** at 80 µM concentration for melan-a cell was over 98.1% and 97.8%, respectively (data not shown). These results clearly indicated compounds **1** and **2** have a non-cytotoxic nature. The effect of anti-melanogenic activities of compounds are shown in Figure 3. The inhibition of melanin synthesis of compound **1** at 20, 40, and 80 µM was 8.4%, 15.6% and 37.0% compared to the control. Compound **2** showed slightly lower inhibitory activity than compound **1** at 7.6%, 12.8% and 17.8% at 20, 40, and 80 µM, respectively. Both compounds inhibited melanin synthesis in a dose-dependent manner. Notably, compound **1** showed the highest melanin inhibitory activity, 37.0% at 80 µM concentration. Reportedly, extract of radix ginseng at 0–1000 µg/mL did not exhibit any significant inhibition of melanin [19], and cinnamic acid, a whitening agent mainly found in *P. ginseng*, showed 29% inhibition of melanin synthesis at 675 µM [20]. Compared with the extract of radix ginseng and cinnamic acid, compound **1** showed potent melanin synthesis inhibitory activity, and even showed a 1.2-fold higher inhibitory activity of melanin synthesis at eight-fold lower concentration compared with cumaric acid [17,20].

**Figure 4:**
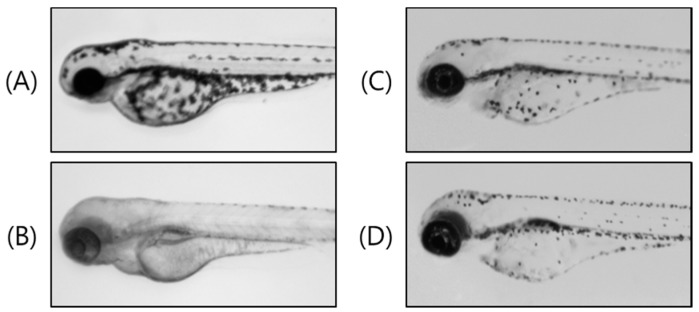
Effects of compound **1** on melanogenesis in zebrafish. Synchronized embryos were treated with melanogenic inhibitors at the indicated concentrations. Compound **1** was dissolved in 0.1% DMSO and then added to the embryo medium. The effects on pigmentation of zebrafish were observed under the stereomicroscope: (**A**) (vehicle control) untreated zebrafish embryo; (**B**) (positive control) 100 µM 1-phenyl-2-thiourea (PTU); (**C**) 40 µM compound **1**; and (**D**) 80 µM compound **1**.

The zebrafish is a highly advantageous vertebrate model organism because of its similar organ systems and gene sequences to human beings [25]. Furthermore, the use of zebrafish embryos is receiving increasing attention since they are considered a replacement method for animal experiments [26]. Zebrafish have melanin pigments on the surface, allowing simple observation of the pigmentation process without complicated experimental procedures [27]. Thus, we investigated the melanin inhibitory effects of compound **1** on the pigmentation of zebrafish. We used PTU (*N*-phenylthiourea; a sulfur-containing tyrosinase inhibitor) as a positive control (Figure 4B), which is used widely in zebrafish research [28,29]. As shown in Figure 4C,D, 40 and 80 µM treatment with compound **1** produced remarkable inhibition of the zebrafish body pigmentation, which remarkably decreased the total melanin content compared with the control vehicle (Figure 4A).

In this study, we isolated novel ginsenoside Rh23 (**1**) from hydroponic *P. ginseng* leaves. Thus far, more than 100 ginsenosides have been reported from ginseng species. However, 25-hydroxylated ginsenosides rarely occur in nature, including in ginseng plants. In addition, whitening activity had not been reported. The inhibitory activity of ginsenoside Rh23 showed the 37% at the 80 µM concentration without cell cytotoxicity in melan-a cells, whereas no inhibition of in vitro mushroom tyrosinase activity was observed by ginsenoside Rh23 (data not shown). Recently, the extracts or purified ginsenosides from ginseng roots and leaves have been shown to widely possess anti-oxidant properties [30,31], while water or organic extract of ginseng has exhibited scavenging activities towards DPPH, superoxide anion and hydroxyl radical [32]. Therefore, our novel ginsenoside Rh23 isolated from the leaves of hydroponic *P. ginseng* may have down-regulated tyrosinase by its anti-oxidative property. However, its melanogenesis role has not been clearly investigated yet. Therefore, in further study, it is necessary to determine the precise mechanisms of ginsenoside Rh23 action on the regulation of melanin synthesis.

## 3. Experimental

### 3.1. General

Kieselgel 60 and LiChroprep RP-18 resins were used for column chromatography (Merck, Darmstadt, Germany). Kieselgel 60 F_254_ (Merck) and RP-18 F_254S_ (Merck) were used as solid phases for TLC experiment. Detection of spots on the TLC plate was performed by observation under a UV lamp (Spectroline, model ENF-240 C/F, Spectronics Corp., New York, NY, USA) or by spraying 10% aqueous H_2_SO_4_ on the developed plate followed by heating. Optical rotations were measured using a JASCO P-1010 digital polarimeter (Tokyo, Japan). Melting points were obtained using a Fisher-Johns Melting Point Apparatus (Fisher scientific company, Pittsburgh, PA, USA) with a microscope. Ultraviolet spectra were measured on a Shimadzu model UV-1601 spectrophotometer (Shimadzu Corp., Kyoto, Japan). IR spectra were obtained from a Perkin Elmer Spectrum One FT-IR spectrometer (Buckinghamshire, UK). NMR spectra were recorded on a Varian Inova AS 400 spectrometer (400 MHz, Varian, Palo Alto, CA, USA). UPLC-QTOF/MS analysis was performed using a Waters Xevo G2-S series (Waters Corp., Milford, MA, USA) operating in the negative ion mode.

### 3.2. Plant Materials

Hydroponic *Panax ginseng* was grown in the greenhouse of the Department of Herbal Crop Research located in Eumseong, Chungbuk Province according to the protocol of “ginseng GAP standard cultivation guide” [11,33] developed by the Rural Development Administration, Republic of Korea. One-year-old ginseng seedling roots weighing 0.8 to 1 g were purchased from Department of Herbal Crop Research, National Institute of Horticultural and Herbal Science (NIHHS), Rural Development Administration (RDA) and stored in a chamber at low temperature (1–2 °C) before use. The ginseng seedling roots were transplanted to nutrient baths and cultured in the hydroponic system. After three months of culture, the hydroponic ginsengs were pulled out for harvesting. The harvested hydroponic ginseng plants were washed clean of dust with water and sorted into leaves and roots, which were then dried for 72 h in a freeze dryer (FD8512, Ilshin Biobase Co., Yangju, Korea). Voucher specimen (NIHHS14-03) was deposited at the herbarium of the Department of Herbal Crop Research, NIHHS, RDA, Eumseong, Republic of Korea.

### 3.3. Extraction and Isolation

The dried and powdered leaves of hydroponic *P. ginseng* (HPGL, 6 kg) were extracted with 80% MeOH (30 L × 3) at room temperature for 24 h. The extracts were filtered through filter paper and evaporated under reduced pressure at 45 °C to yield 1.4 kg of extract. The extract was poured into H_2_O (3L) and extracted with EtOAc (3 L × 3) and *n*-BuOH (2.6 L × 3), successively. Each layer was concentrated under reduced pressure to obtain EtOAc (75 g), *n*-BuOH (470 g), and H_2_O (855 g) fractions. The *n*-BuOH fraction (HPGLB, 130 g) was applied to the silica gel column (φ 13 × 17 cm) and eluted with CHCl_3_–MeOH–H_2_O (8:3:1, 90 L → 6:4:1, 110 L) to yield 20 fractions (HPGLB1 to HPGLB20). Fractions HPGLB3 and HPGLB4 were combined (18.9 g, Ve/Vt = 0.05–0.12), and further fractionated over the silica gel column (φ 8 × 15 cm, CHCl_3_–MeOH–H_2_O = 12:3:1, 14 L) to yield 14 fractions (HPGLB3-1 to HPGLB3-14). Fractions HPGLB3-4 and HPGLB3-5 were combined (1.27 g, Ve/Vt = 0.09–0.16), and further fractionated over the ODS column (φ 4 × 7 cm, MeOH–H_2_O = 2:1, 2.6 L) to yield nine fractions (HPGLB3-4-1 to HPGLB3-4-9). Fraction HPGLB3-4-3 (119.4 mg, Ve/Vt = 0.14–0.26) was further fractionated over the ODS column (φ 2.5 × 7 cm, MeOH–H_2_O = 1:1, 1 L) to yield nine fractions (HPGLB3-4-3-1 to HPGLB3-4-3-9) including compound **1** (HPGLB3-4-3-7, 10.5 mg, Ve/Vt = 0.42–0.55, TLC R_f_ = 0.40 (RP-18 F_254S_, MeOH–H_2_O = 2:1), R_f_ = 0.50 (Kieselgel 60 F_254_, CHCl_3_–MeOH–H_2_O = 7:3:1)). Fractions HPGLB5 to HPGLB7 were combined (24.0 g, Ve/Vt = 0.12–0.22), and further fractionated over the silica gel column (φ 7 × 12 cm, CHCl_3_–MeOH–H_2_O = 10:3:1, 10 L → 6:4:1, 9 L) to yield 16 fractions (HPGLB5-1 to HPGLB5-16). Fraction HPGLB5-6 (323.1 mg, Ve/Vt = 0.28–0.31) was further fractionated over the ODS column (φ 3 × 14 cm, MeOH–H_2_O = 3:2, 1.2 L → 3:1, 1.5 L) to yield ten fractions (HPGLB5-6-1 to HPGLB5-6-10) including compound **2** (HPGLB5-6-5, 10.1 mg, Ve/Vt = 0.21–0.23, TLC R_f_ = 0.50 (RP-18 F_254S_, MeOH–H_2_O = 2:1), R_f_ = 0.45 (Kieselgel 60 F_254_, CHCl_3_–MeOH–H_2_O = 7:3:1)).

### 3.4. Spectroscopic Data

Compound **1**. White powder, m.p.: 138–140 °C; $[\alpha {]}_{D}^{25}$ +17.4° (*c* = 0.39, MeOH); IR (CaF_2_ window): 3377, 2932, 1382 cm^−1^; negative QTOF/MS *m*/*z* 713.44723 [M + COOH]^−^ (calcd. for C_37_H_64_O_10_, 668.4499); ^1^H- and ^13^C-NMR data, see Table 1.

Compound **2**. White powder, m.p.: 133–136 °C; $[\alpha {]}_{D}^{25}$ +20.2° (*c* = 0.50, MeOH); IR (CaF_2_ window): 3359, 2929, 1384 cm^−1^; negative QTOF/MS *m*/*z* 699.48311 [M + COOH]^−^ (calcd. for C_36_H_62_O_10_, 654.4323);^1^H- and ^13^C-NMR data, see Table 1.

### 3.5. Quantitative Analysis of Novel Compound ***1*** Using UPLC-QTOF/MS

Standard stock solution of compound **1** was prepared by dissolving 1.00 mg each in 1 mL methanol to yield a concentration of 1.00 mg/mL and were kept at 4 °C. The standard stock solution (**1**) was diluted with methanol to obtain calibration solutions with ranges of 0.02–0.8 µg/mL, respectively. One gram of HPGL extract was accurately weighed and dissolved in fixed volumes (10 mL) of methanol, filtered through a 0.20 mm filter paper, and refrigerated at 4 °C. UPLC was performed using a Waters ACQUITY H-Class UPLC (Waters Corp., Milford, MA, USA) with an ACQUITY BEH C18 column (2.1 × 100 mm, 1.7 μm). The mobile phases consisted of water (A) with 0.1% formic acid (*v*/*v*) and acetonitrile (B) with 0.1% formic acid (*v*/*v*). The elution gradient was as follows: 0–4 min, B 10–30%; 4–15 min, B 30–60%; 15–16 min, B 60–100%; 16–19 min, B 100–10%. The flow rate was 0.45 mL/min, and the injection volume was 2 μL for each run. Next, HR-MS analysis was performed using a Waters Xevo G2-S QTOF MS (Waters Corp., Milford, MA, USA) operating in negative ion mode. The mass spectrometers performed alternating high- and low-energy scans known as MS^E^ acquisition mode. Accurate mass measurements were obtained by means of an automated calibration delivery system containing Leucine enkephalin, *m/z* 554.262 (ESI neg. mode) as an internal reference. Optimal operating parameters were set as shown in Table 3.

### 3.6. Cell Culture

Melan-a melanocytes are a highly-pigmented, immortalized, normal murine melanocyte cell line derived from C57BL/6 mice. The melan-a cells used in this study were obtained from Dr. Dorothy Bennett (St. George’s Hospital, London, UK). Cells were cultured at 37 °C in an atmosphere of 95% air, 10% CO_2_ in RPMI 1640 medium supplemented to a final concentration with 10% heat-inactivated fetal bovine serum, 1% penicillin/streptomycin, and 200 nM PMA. Cell viability was determinate by CCK-8 cell counting kit-8 (Dojindo Lab., Kumamoto, Japan).

### 3.7. Melanin Assay

Melan-a cells were treated with compounds for 72 h, and then the cells were dissolved in 1 N NaOH at 60 °C for 30 min. Then, the lysates were measured at 450 nm using a spectrophotometer. The data were normalized to the protein content of the cell lysates. The cell lysates were subsequently processed for the determination of the protein concentration using a BCA protein assay kit (Thermo Fisher Scientific Inc., Rockford, IL, USA).

### 3.8. Origin and Maintenance of Parental Fish

Adult zebrafishes were obtained from a commercial dealer and 10–15 fishes were kept in a 5 L acrylic tank with the following conditions: 28.5 °C, with a 14/10 h light/dark cycle. Zebrafishes were fed three times a day, six days per week, with TetraMin flake food supplemented with live brine shrimps (*Artemia salina*). Embryos were obtained from natural spawning that was induced at the morning by turning on the light. Collection of embryos was completed within 30 min. All experimental protocols and procedures were approved and conducted according to the approved guidelines and regulations of the Animal Ethics Committee of Chungnam National University (CNU-00866).

### 3.9. Compounds Treatment and Phenotype-Based Evaluation

Synchronized embryos were collected and arrayed by pipette (7–9 embryos per well in 24-well plates containing 1 mL of embryo medium). Test compounds were dissolved in 0.1% DMSO, and then added to the embryo medium from nine to 72 h post-fertilization (h.p.f) (63 h of exposure). The effects on the pigmentation of zebrafish were observed under the stereomicroscope. Occasional stirring, as well as a replacement of the medium, was performed daily to ensure the even distribution of the compounds. In all experiments, 0.2 mM 1-phenyl-2-thiourea (PTU) was used to generate transparent zebrafish without interfering in the developmental process [33], and was considered as a standard positive control. Phenotype-based evaluations of body pigmentation were dechorionated by forceps, anesthetized in tricaine methanesulfonate solution (Sigma-Aldrich, St. Louis, MO, USA), mounted in 3% methylcellulose on a 35 mm dish (SPL Lifesciences, Pocheon, Korea), and photographed under the stereomicroscope MZ16 (Leica Microsystems GmbH, Wetzlar, Germany).

## 4. Conclusions

In this study, ginsenoside Rh23 (**1**) was isolated from hydrophonic *Panax ginseng* leaves together with 20-*O*-β-d-glucopyranosyl-3β,6α,12β,20β,25-pentahydroxydammar-23-ene (**2**). We have been generally successful in our attempt to obtain the hypopigmentary effect of compounds from hydroponic *P. ginseng.* Ginsenoside Rh23 and 20-*O*-β-d-glucopyranosyl-3β,6α,12β,20β,25-pentahydroxydammar-23-ene have inhibitory activities on melanin biosynthesis without cytotoxic effects in melan-a cell. Additionally, ginsenoside Rh23 enhanced the depigmentation on the zebrafish as an alternative animal model. The decrease of melanin content and pigmentation on the body may have the potential for whitening activity and non-cytotoxic effect is more favorable point because safety is a primary consideration for whitening agent in cosmetic products. Thus, based on our current results, it is important that novel hydrophonic *P. ginseng* compounds can be used as a potential effective skin-lighting agent. Further studies will elucidate the precise mechanisms of ginsenoside Rh23 action on the regulation of melanin synthesis and the relationship between structural characteristics of ginsenoside Rh23 and melanogenesis, to ascertain whether it is a potential whitening agent for the cosmetic industry. The advantages of hybrid Q-TOF mass spectrometry include not only quality detection capability and sensitivity, but also accurate measurement, making structural elucidations easier. It can be used for qualitative and quantitative determination of minor or novel compounds, which is helpful in improving the quality control of ginseng spices.

## Figures and Tables

**Table 1:** ^1^H- (400 MHz) and ^13^C-NMR (100 MHz) spectra of compounds **1** and **2** (in pyridine-*d*_5_).

	Compound 1		Compound 2	
No.	δ_H_ in ppm, *J* in Hz	δ_C_	δ_H_ in ppm, *J* in Hz	δ_C_
1	1.73, 1.02	39.4	1.70, 1.00	39.5
2	1.84, 1.83	28.1	1.83, 1.81	28.1
3	3.49 (1H, dd, *J* = 11.6, 4.8 Hz)	78.5	3.50 (1H, dd, *J* = 11.2, 5.2 Hz)	78.5
4	-	40.3	-	40.3
5	1.21 (1H, d, *J* = 10.4 Hz)	61.8	1.20 (1H, d, *J* = 10.4 Hz)	61.8
6	4.38 (1H, m)	67.7	4.37 (1H, m)	67.7
7	1.92, 1.87	47.4	1.91, 1.86	47.4
8	-	41.2	-	41.2
9	1.54	49.3	1.53	49.8
10	-	39.4	-	39.3
11	2.11, 1.58	31.0	2.08, 1.53	31.0
12	4.03 (1H, m)	70.3	4.03 (1H, m)	70.4
13	1.97 (1H, m)	49.3	1.97 (1H, m)	49.2
14	-	51.4	-	51.4
15	1.61, 0.99	30.7	1.57, 0.99	30.6
16	1.74, 1.40	26.4	1.72, 1.41	26.4
17	2.45 (1H, m)	52.1	2.40 (1H, m)	52.4
18	1.14 (3H, s)	17.6	1.13 (3H, s)	17.6
19	1.04 (3H, s)	17.4	1.03 (3H, s)	17.4
20	-	83.0	-	83.2
21	1.55 (3H, s)	23.0	1.55 (3H, s)	22.9
22	3.07 (1H, dd, *J* = 14.0, 5.6 Hz) 2.64 (1H, dd, *J* = 14.0, 8.8 Hz)	39.6	3.01 (1H, dd, *J* = 14.0, 6.0 Hz) 2.71, (1H, dd, *J* = 14.0, 8.8 Hz)	39.3
23	6.02 (1H, ddd, *J* = 15.6, 8.8, 5.6 Hz)	126.8	6.26 (1H, ddd, *J* = 15.6, 8.8, 6.0 Hz)	122.8
24	5.64 (1H, d, *J* = 15.6 Hz)	138.5	5.96 (1H, d, *J* = 15.6 Hz)	142.0
25	-	74.9	-	69.0
26	1.31 (3H, s)	26.1	1.51 (3H, s)	30.6
27	1.33 (3H, s)	26.3	1.50 (3H, s)	30.8
28	1.94 (3H, s)	31.9	1.95 (3H, s)	31.9
29	1.43 (3H, s)	16.4	1.42 (3H, s)	16.4
30	0.92 (3H, s)	17.3	0.89 (3H, s)	17.2
OCH_3_	3.18 (3H, s)	50.2	-	-
1′	5.15 (1H, d, *J* = 7.6 Hz)	98.3	5.15 (1H, d, *J* = 7.6 Hz)	98.4
2′	3.95 (1H, dd, *J* = 8.8, 7.6 Hz)	75.2	3.94 (1H, dd, *J* = 8.4, 7.6Hz)	75.2
3′	4.18 (1H, dd, *J* = 8.8, 8.8 Hz)	78.9	4.14 (1H, dd, *J* = 8.8, 8.4 Hz)	78.9
4′	4.09 (1H, dd, *J* = 9.6, 8.8 Hz)	71.6	4.07 (1H, dd, *J* = 9.6, 8.8 Hz)	71.7
5′	3.90 (1H, ddd, *J* = 9.6, 5.6, 2.4 Hz)	78.2	3.90 (1H, ddd, *J* = 9.6, 5.6, 2.4 Hz)	78.2
6′	4.45 (1H, dd, *J* = 11.6, 2.4 Hz) 4.27 (1H, dd, *J* = 11.6, 5.6 Hz)	63.0	4.46 (1H, dd, *J* = 11.6, 2.4 Hz) 4.23 (1H, dd, *J* = 11.6, 5.6 Hz)	62.9

**Table 2:** Linear regression data and contents of the validated method for the ginsenoside Rh23 in HPGL extract ^a^.

Compounds	R_t_^b^ (min)	Calibration Curve ^c^	R^2^	Line Arrange (µg/mL)	LOD ^d^ (ppm)	LOQ ^e^ (ppm)	Amount (mg/g)
Rh23	10.79	*y* = 351*x* + 34.9	0.996	0.02–0.8	0.002	0.005	0.319

^a^ Mean values of samples (*n* = 3). ^b^ Rt, retention time. ^c^ *y*, logarithmic value of peak area; *x*, logarithmic value of amount injected. ^d^ LOD, limit of detection. ^e^ LOQ, limit of quantification.

**Table 3:** Optimal conditions for the Q-TOF/MS analysis of HPGL extract.

Optimal Q-TOF/MS Condition	
Ion Source	ESI Negative Mode
Source Temp. and Desolvation Temp.	120 °C/550 °C
Cone Gas Flow and Desolvation Gas Flow	30 L/h/800 L/h
Capillary Volt and Cone Volt	3 k/40 V
Mass Range (*m*/*z*)	50 to 1000
Collision Energy Range	4 to 45 eV

## Supplementary Materials

^1^H-NMR and ^13^C-NMR spectra of **1** are available in the Supplementary Materials.
