# Supplementary figures and images for: Isolation and Quantification of Ginsenoside Rh23, a New Anti-Melanogenic Compound from the Leaves of Panax ginseng
Source: Molecules. 2018 Jan 29;23(2):267. doi: 10.3390/molecules23020267 (PMC6017343; doi:10.3390/molecules23020267)

Supplementary Materials

<sup>1</sup>H-NMR data of ginsenoside Rh23

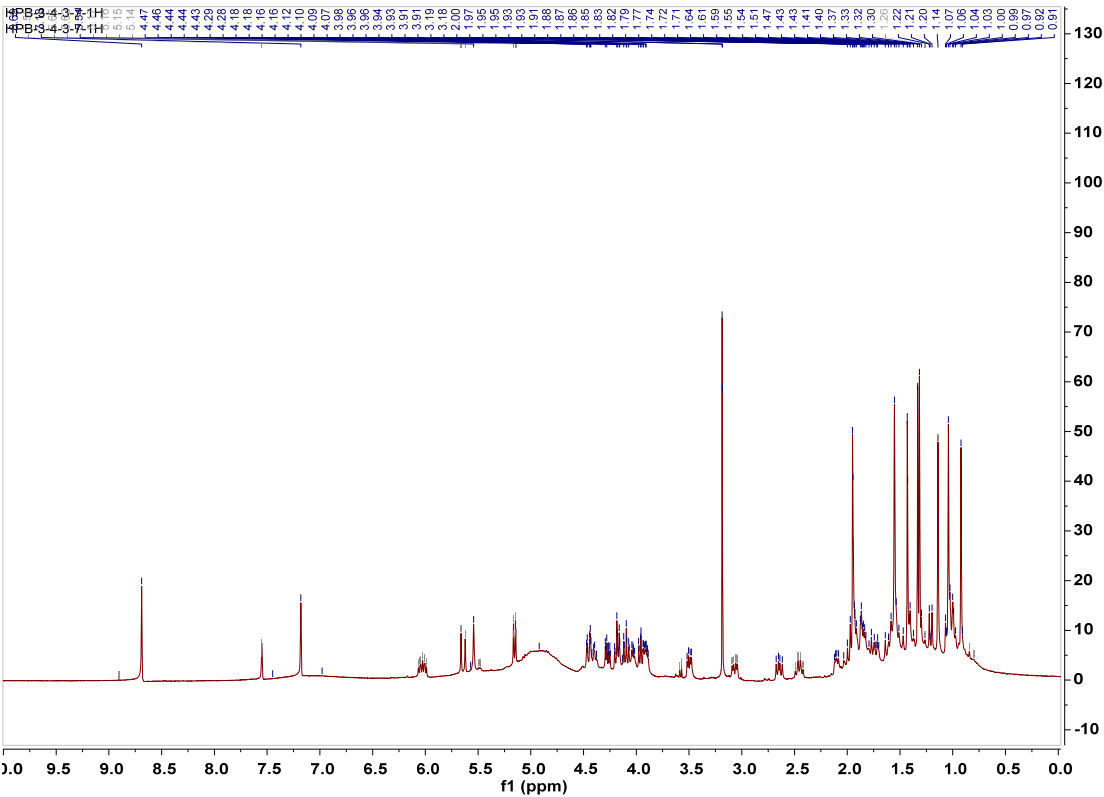

$^{13}\text{C}$ -NMR data of ginsenoside Rh23

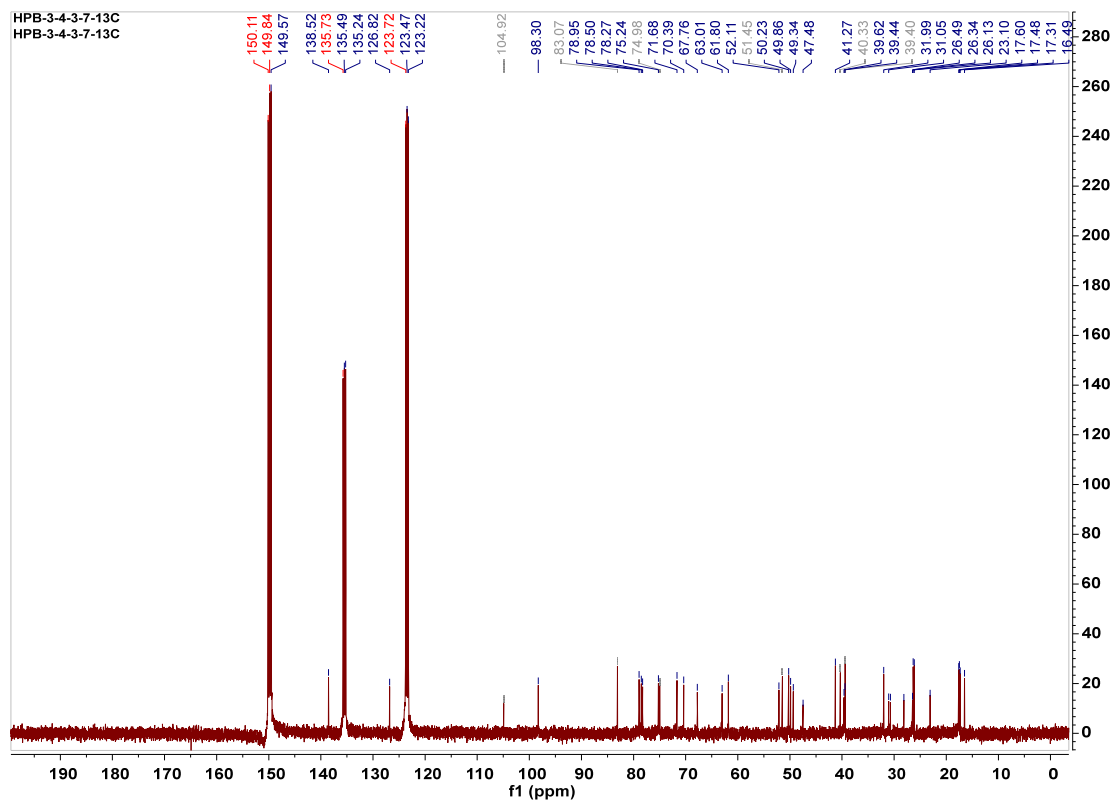

Supplement: Supplementary file 1 [file molecules-23-00267-s001.pdf]
